# *Rgcs1*, a dominant QTL that affects retinal ganglion cell death after optic nerve crush in mice

**DOI:** 10.1186/1471-2202-9-74

**Published:** 2008-07-31

**Authors:** Joel A Dietz, Yan Li, Lisa M Chung, Brian S Yandell, Cassandra L Schlamp, Robert W Nickells

**Affiliations:** 1 Department of Ophthalmology and Visual Sciences, University of Wisconsin, Madison, WI, USA; 2 Department of Biostatistics and Medical Informatics, University of Wisconsin, Madison, WI, USA; 3 Department of Physiology, University of Wisconsin, Madison, WI, USA

## Abstract

**Background:**

Intrinsic apoptosis of neuronal somas is one aspect of neurodegenerative diseases that can be influenced by genetic background. Genes that affect this process may act as susceptibility alleles that contribute to the complex genetic nature of these diseases. Retinal ganglion cell death is a defining feature of the chronic and genetically complex neurodegenerative disease glaucoma. Previous studies using an optic nerve crush procedure in inbred mice, showed that ganglion cell resistance to crush was affected by the Mendelian-dominant inheritance of 1–2 predicted loci. To assess this further, we bred and phenotyped a large population of F2 mice derived from a resistant inbred strain (DBA/2J) and a susceptible strain (BALB/cByJ).

**Results:**

Genome wide mapping of the F2 mice using microsatellite markers, detected a single highly significant quantitative trait locus in a 25 cM (58 Mb) interval on chromosome 5 (Chr5.loc34-59 cM). No interacting loci were detected at the resolution of this screen. We have designated this locus as Retinal ganglion cell susceptible 1, *Rgcs1*. *In silico *analysis of this region revealed the presence of 578 genes or expressed sequence tags, 4 of which are highly expressed in the ganglion cell layer of the mammalian retina, and 2 of which are suspected susceptibility alleles in chronic neurodegenerative diseases. In addition, 25 genes contain 36 known single nucleotide polymorphisms that create nonsynonymous amino acid changes between the two parental strains. Collectively, this analysis has identified 7 potential candidate genes that may affect ganglion cell death.

**Conclusion:**

The process of ganglion cell death is likely one of the many facets of glaucoma susceptibility. A novel dominant locus has been identified that affects sensitivity of ganglion cells to optic nerve crush. The allele responsible for this sensitivity may also be a susceptibility allele for glaucoma.

## Background

Glaucoma is a blinding disease characterized by the progressive death of retinal ganglion cells. The principal risk factor for glaucoma is elevated intraocular pressure (IOP) [1-3]. Biomechanical engineering studies suggest that IOP-related stress is focused on ganglion cell axons exiting the eye through the lamina cribrosa [4,5]. Current models suggest that optic nerve glia are adversely affected and that this leads first to destruction of the ganglion cell axon, and secondarily, to the apoptotic death of the ganglion cell soma (reviewed by [6,7]).

Glaucoma is a complex genetic disease [8]. After elevated IOP, family history is the next most important risk factor [9,10]. While many important studies have revealed a great deal about the genetics of this disease, the majority of these have been restricted to relatively rare forms that exhibit some form of Mendelian inheritance and for which there are large pedigrees that contribute both phenotypic and genotypic information. For most forms of glaucoma, there is limited understanding of the genetics underlying disease susceptibility. One approach to identifying glaucoma susceptibility alleles is to first identify candidate genes using mouse genetics. Specifically, we hypothesized that the process of retinal ganglion cell death may be affected by genetic background. Several studies in mice have documented quantitative trait loci (QTL) that associate with neuronal degeneration. These include loci that affect neuronal susceptibility in the substantia nigra to the Parkinsonian drug MPTP [11,12] or sensitivity to kainic acid in the hippocampus [13]. Additionally, reduced *Bax *expression in ganglion cells created in gene dosage experiments, completely abrogates soma death in both acute optic nerve lesion and chronic glaucoma models in mice [14,15].

In an effort to identify alleles affecting ganglion cell death, we screened inbred mice to examine if genetic background influenced cell loss after optic nerve crush. This lesion stimulates several of the same molecular pathways active in dying ganglion cells in glaucoma [15-19]. A screen of 15 different lines showed that genetic background did affect the loss of these cells. Reciprocal backcross breeding experiments using the most resistant (DBA/2J) and susceptible lines (BALB/cByJ) indicated that the resistant phenotype was attributable to 1–2 dominant loci [20]. In this report, we have extended this observation to show the results of a genome wide screen of a large F2 mapping population generated from these two parental inbred lines. This study reveals a single significant dominant QTL on chromosome 5 that associates with the cell death phenotype. We have designated this locus as Retinal ganglion cell susceptible 1 (*Rgcs1*).

## Results

### A single dominant QTL affecting retinal ganglion cell death is located on chromosome 5

**Figure 1:**
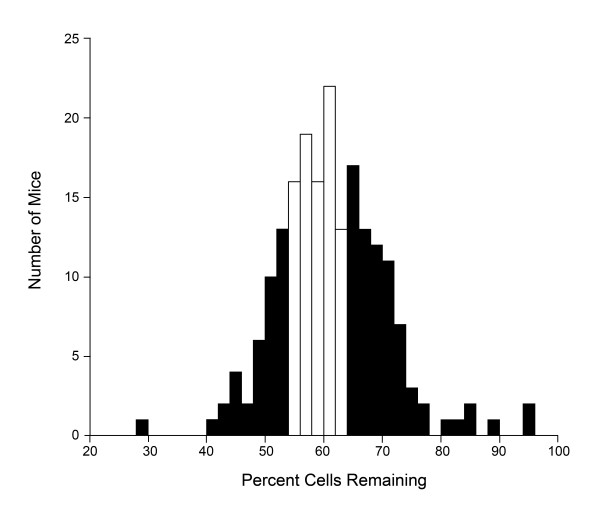
**Frequency histograph of phenotype for the 196 mice in the F2 mapping population**. The loss of cells in each experimental retina was quantified as a percentage of cells present in the control retina of each mouse. Filled bars indicate mice in each tail of the population distribution that were selected for genotyping. Susceptible mice exhibited ≤ 54% cells remaining, while resistant mice exhibited ≤ 64% cells remaining.

A population of 196 F2 mice underwent optic nerve crush and cell loss phenotyping. The distribution of phenotypes in this population (Fig. 1) matched well with a smaller population of F2 mice examined previously [20], indicating that the phenotype was consistently inherited. For genome wide mapping, we selected mice from the F2 population that had 54% or less cells remaining (the most susceptible mice) and 64% or more cells remaining (the most resistant mice). These animals are indicated by the filled bars in the phenotype frequency histograph of the F2 population (Fig. 1).

**Figure 2:**
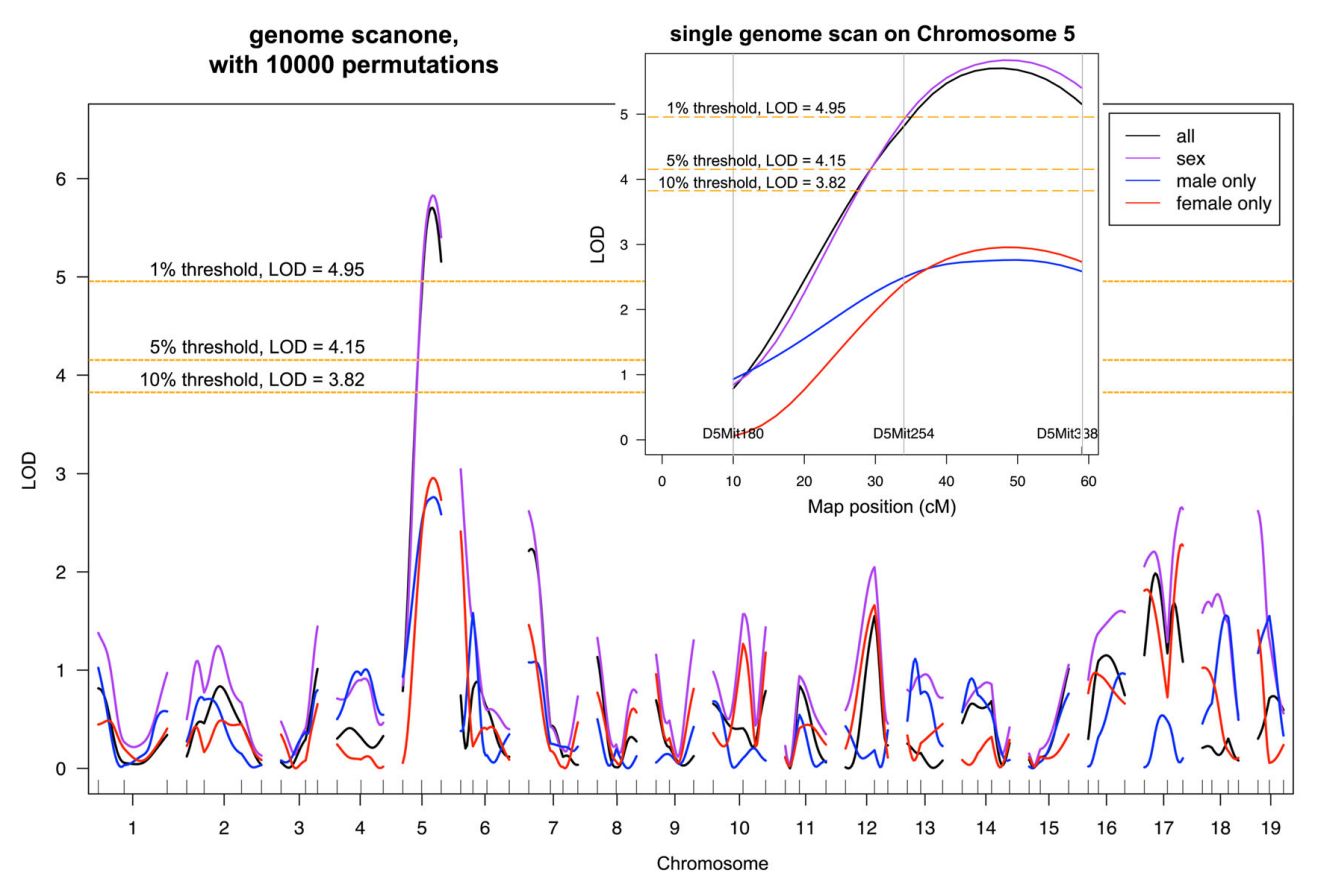
**A genome scan showing a significant QTL on chromosome 5 that maps to the cell death phenotype**. The microsatellite mapping data was analyzed individually for male and female mice, and for all the mice with and without sex as an interactive covariate. The observed LOD score was compared to 10000 LOD scores with permuted phenotypes [61], yielding a 5% significance threshold of 4.15. The inset shows a detail of the LOD scores for all 3 markers used for chromosome 5 (Chr5). The region of interest maps between 34 and 59 cM with the predicted maximum LOD score (5.825) at 38 cM. This locus has been designated as Retinal ganglion cell susceptible 1 (*Rgcs1*).

Quantitative trait linkage analysis using 65 microsatellite markers, with and without sex as a covariate, showed a single significant peak on chromosome 5 between markers D5Mit254 (34 cM) and D5Mit338 (59 cM) (Fig. 2). The maximum LOD score predicted by interval analysis was 5.825 at Chr5.loc38 (inset Fig. 2), which was the only locus that exceeded the significance level of *P *= 0.05 (*P *= 0.0011 for the peak). The next highest LOD score in the analysis of all the samples modeled without interactive covariates was 3.045 for D6Mit83 on chromosome 6 (Chr6.loc3.5). It is notable that evaluation of larger numbers of animals from the F2 population may have not only increased the significance of the locus on chromosome 5, but also increased the LOD score at Chr6.loc3.5 to above the threshold value. The QTL dataset was also analyzed for interacting loci, again including sex as a covariate. No significant associations were detected at the resolution of this genome-wide screen, thus a single locus on chromosome 5 accounts for 11.61% of the phenotypic variance of this trait. This locus has been designated as Retinal ganglion cell susceptible 1 (*Rgcs1*). For subsequent analyses, we have conservatively defined this locus by the nearest markers used for mapping. This 58 Mb region includes an approximation of the 95% confidence interval as suggested by a 1.5 LOD drop from the peak [21].

**Figure 3:**
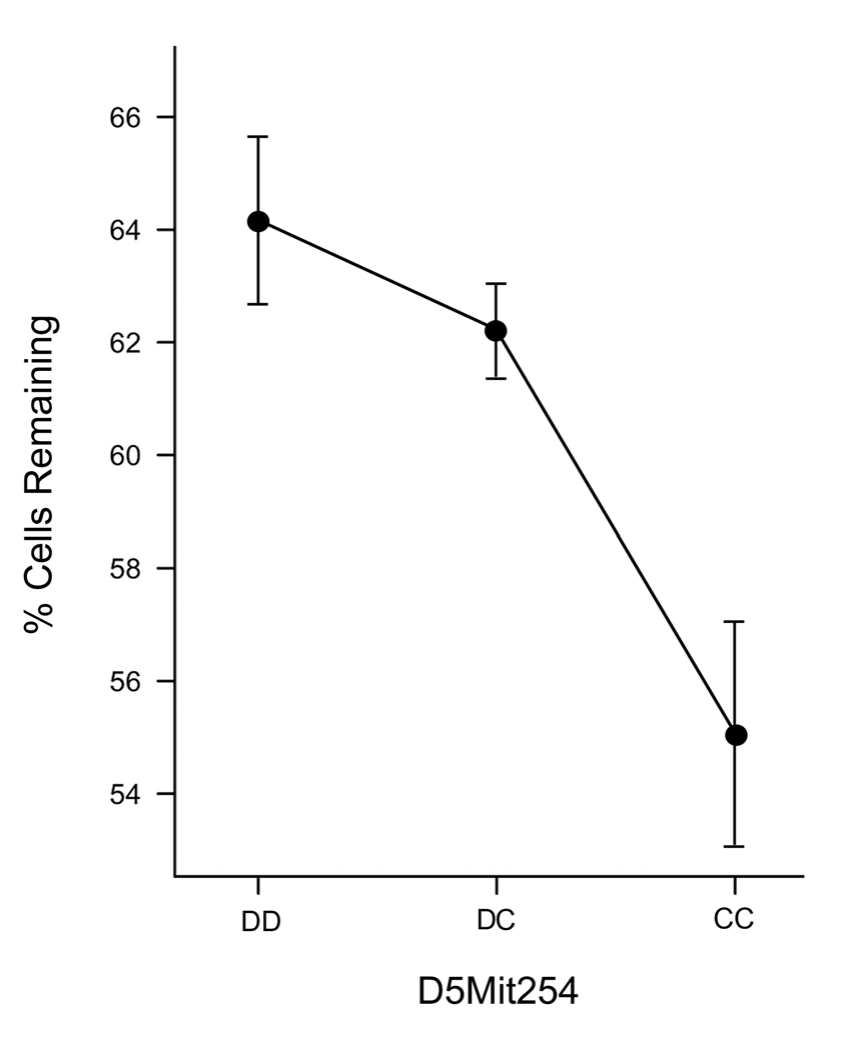
***Rgcs1 *is a Mendelian-dominant locus affecting ganglion cell susceptibility to optic nerve crush**. The mean (± s.d.) cells remaining for F2 mice were plotted as a function of inheritance of the D5Mit245 polymorphic marker. This marker is closest to the predicted location of chromosome 5 with the highest LOD score. Mice homozygous or heterozygous for the DBA/2J allele (D) have statistically equivalent phenotypes (*t*-test, *P *= 0.169), while mice that have inherited 2 BALB/c alleles (C) have significantly fewer cells remaining compared to F2 mice carrying a D allele (*P *= 4.4 × 10^-7^). These C/C mice exhibit a phenotype that is similar to the susceptible phenotype of the BALB/c parental strain.

We also examined the inheritance pattern of phenotype as a function of marker D5Mit254, which lies closest to the region with the statistical maximum LOD score (Fig. 3). Mice inheriting 2 BALB/c alleles have significantly fewer cells remaining than mice inheriting 1 or 2 DBA/2J alleles (*t*-test, *P *= 4.4 × 10^-7^). Conversely, mice heterozygous for the DBA/2J locus exhibited a statistically equivalent phenotype to mice homozygous at this locus (*P *= 0.169). Together, these data are supportive of our original observation of the Mendelian-dominant nature of the *Rgcs1 *locus obtained from reciprocal breeding experiments [20].

### Identification of candidate genes in the Rgcs1 locus

Genomic sequence information, available for multiple different strains of mice (please see Availability & requirements for more information), was examined to identify candidate genes. The region of Chr5.loc34-59 was found to be relatively gene poor, with a total of 578 known genes and/or expressed sequence tags (ESTs) identified. Based on existing sequence data, we used *in silico *analysis to find single nucleotide polymorphisms (SNPs) creating non-synonymous amino acid changes between the DBA/2J and BALB/cByJ strains. This search identified 36 amino acid changes in 25 genes (Table 1). Of these changes, 12 were conservative changes and 24 were non-conservative, including 2 that change a hydrophobic amino acid in DBA/2J mice to a proline in BALB/c mice (genes *Tlr1 *and *Ugt2a3*).

**Table 1:** Summary of SNP polymorphisms between DBA/2J and BALB/c mice, affecting coding regions of genes and ESTs in the *Rgcs1 *locus

Gene Name	DBA/2J	AA	BALB/c	AA	Position	Conserved*	Strain Cluster^†^
Tlr1	C	Q	T	R	667	No	Possible
*Tlr1*	G	L	A	P	246	No	Possible
*Tlr1*	G	L	A	S	111	No	No
*Tlr6*	T	V	C	I	681	Yes	Yes
*9130005N14Rik*	G	T	A	A	498	No	No
*Recc1*	C	N	T	S	588	Yes	No
*Klb*	T	T	C	M	511	No	No
*B3bp*	A	S	G	G	1626	No	No
*EG545758*	A	S	G	L	141	No	No
*Npal1*	G	H	A	R	178/197	Yes	No
*BC031901*	C	G	T	E	432	No	No
*BC031901*	A	R	C	L	398	No	No
*EG627807*	T	S	C	N	44	Yes	No
*Rest*	A	G	G	S	555	No	No
*EG384187*	C	E	G	Q	67	No	No
*EG384187*	G	V	T	G	91	Yes	No
*EG384187*	T	K	A	N	144	No	No
*EG384187*	G	V	T	G	160	Yes	No
*EG384187*	T	T	C	I	164	No	No
*Ugt2b1*	G	L	A	F	425	No	No
*Ugt2a3*	T	T	G	P	508	No	Possible
*Ugt2a2*	G	A	T	E	500	No	N/A
*Ugt2a2*	C	R	G	G	467	No	Yes
*C230008H04Rik*	C	K	T	E	37	No	No
*Slc10a6*	C	T	T	A	2	No	No
*Klhl8*	T	R	C	Q	18	No	No
*Sparcl1*	G	R	A	Q	384	No	Possible
*Zfp326*	G	G	A	D	493	No	Possible
*Zfp644*	C	N	T	S	210	Yes	No
*4921521K07Rik*	A	I	G	V	102	Yes	Possible
*4921521K07Rik*	T	F	A	Y	280	Yes	No
*A830010M20Rik*	G	D	A	N	382/630	No	No
*A830010M20Rik*	G	S	A	N	762/1010	Yes	Possible
*AW060207*	G	D	A	G	279	No	Possible
*AW060207*	G	H	C	Q	355	Yes	No
*Pigg*	G	V	A	I	132	Yes	No

*Conservative vs Non-conservative amino acids (AA) were assigned based on differences in charge and hydrophobicity. †Strain clustering refers to data showing the same SNP in at least one reference mouse strain with a similar quantitative phenotype. A score of No indicates a DBA/2J or BALB/c polymorphism in at least one conflicting reference strain. A score of Possible indicates polymorphisms that fit trend for intermediate strains, but for which there is limited data for reference strains, or where there is data only for one set of reference strains. Reference strains examined were 129X1/SVJ and C57Bl/6J for DBA/2J mice, and NOD/LtJ and C3H/HeJ for BALB/cByJ mice. Intermediate strains were A/J and NZB/BINJ [20].

We also examined the list of polymorphisms for similarities to other mouse strains with cell death phenotypes similar to either the resistant or susceptible strains. Of the 25 genes identified, 2 genes clearly exhibited strain clustering of a polymorphism, while 7 other genes (totaling 8 SNPs) showed possible clustering. The basis for this latter definition was that only partial sequence data was available for reference strains or intermediate strains (see Methods). Of these 9 potential genes, 7 exhibited non-conservative amino acid changes.

A second layer of analysis was then conducted to identify genes in this region that were highly expressed or enriched in cells of the ganglion cell layer, including ganglion cells themselves, by screening available microarray data. Four genes in the *Rgcs1 *region were found as being either highly enriched, or highly expressed, in the ganglion cell layer and optic nerve head, including *Pcdh7*, *Uchl1*, *Sparcl1*, and *Cplx1 *(Table 2). Of these 4, *Sparcl1 *appeared on both lists of SNP-containing genes and genes expressed in the ganglion cell layer. All 4 of these candidate genes showed a decrease in expression level in arrays of axotomized or glaucomatous rat retinas [22].

**Table 2:** Summary of all candidate genes.

Gene Name	Chromosome Position (starting bp)	Retinal Expression	Coding Region SNP^c^	Strain Association^d^	Role in Neurodegeneration
*Pcdh7*	58006337	GCL enriched^a,b^	No	N/A	Unknown
*Tlr1*	65203969	Confirmed^b^	Yes	Possible	Immune Modulation^e^
*Tlr6*	65232241	Confirmed^b^	Yes	Yes	Immune Modulation^e^
*Uchl1*	66955376	GCL enriched^a,b^	No	N/A	eQTL Susceptibility Allele^f^
*Sparcl1*	104319413	GCL enriched^a,b^	Yes	Possible	Possible Glial Activation^g^
*Cplx1*	108760431	GCL enriched^a,b^	No	N/A	2° Degeneration^h^
*Hspb8*	116669490	Confirmed^b^	No	N/A	Susceptibility Allele^i^

The chromosomal position of each gene is shown, along with a short summary of how each gene scored in the different criteria for identifying candidate genes.

^a^Identified from microarray expression profiles.

^b^The expression of each candidate gene was confirmed in the mouse retina using reverse transcriptase-PCR amplification of a portion of its cDNA (data not shown).

^c^Single nucleotide polymorphism (SNP) analysis was conducted *in silico *using sequence information available on the MGI data base. SNPs that resulted in coding region changes between DBA/2J and BALB/cByJ mice were examined.

^d^Coding region SNPs between DBA/2J and BALB/cByJ mice were compared to reference strains if sequence data were available. Reference strains included 129X1/SVJ and C57Bl/6J as resistant strains comparable to DBA/2J mice, and NOD/LtJ and C3H/HeJ as susceptible strains comparable to BALB/cByJ mice. Intermediate strains included A/J (as a likely resistant strain) and NZB/BINJ (as a likely susceptible strain). For genes showing a SNP difference, if the same SNP was noted in the reference strains it was scored as showing a positive strain association. A possible strain association was recorded if sequence data was limited to only a resistant or susceptible reference strain, or the intermediate strains showed the predicted strain association, but there was not data available for the reference strains. If no SNP was noted, the strain association analysis was not applicable (N/A).

^e^[31,33].

^f ^[34-36].

^g^[37].

^h^[40].

^i^[45]

Finally, a search of the 578 genes was made to identify candidates that had already been identified as susceptibility alleles for other neurodegenerative diseases. This search yielded 2 genes, *HspB8 *and *Uchl1*. The latter gene was also identified in the search of genes present in the retinal ganglion cell layer. Collectively, using the criteria of expression in the retina, ganglion cell layer, or other CNS neurons; and/or the presence of strain-specific non-conserved amino acid changes; and/or the involvement of genes in neurodegeneration, we identified 7 genes as possible candidate genes that could affect retinal ganglion cell death after lesion to the optic nerve (Table 2).

## Discussion

In an attempt to determine if genetic background influences the process of retinal ganglion cell death after damage to the optic nerve, we conducted a screen of inbred mice for susceptibility to a crush lesion of the optic nerve. Breeding studies conducted as part of this series of experiments, suggested that resistance to crush was inherited in a dominant fashion involving relatively few alleles [20]. This finding was expanded to conduct genome wide mapping of a population of F2 mice generated from resistant (DBA/2J) and susceptible (BALB/cByJ) parental strains. Mapping identified a single QTL on chromosome 5, spanning a region of 25 cM delineated by markers D5Mit254 and D5Mit338, which significantly associated with the dominant inheritance of resistance to the crush procedure. *In silico *analysis of the 578 known genes and ESTs in this region identified 7 candidate genes that can be studied further, in concert with more detailed mapping studies to narrow the region of interest.

*Rgcs1 *represents a novel QTL relative to other loci that have been mapped in association with neurodegeneration. These include QTLs on chromosomes 1 [11], 13, and 15 [12], that associate with MPTP toxicity and a QTL on chromosome 18 that affects susceptibility to kainic acid [13]. Interestingly, *Rgcs1 *lies within a QTL that maps between 26 and 61 cM on chromosome 5 that affects the thresholds for electroshock induced seizures in mice [23]. The causative gene is likely not linked to *Rgcs1*, however, since DBA/2J mice exhibit greater susceptibility to electroshock. Lastly, inbred strains of mice exhibit a bimodal distribution of retinal ganglion cell number in adult animals that has been genetically mapped to a QTL on chromosome 1 [24]. Since ganglion cell number in adults is influenced by the process of cell death during development, this region has been examined for its effects on both ganglion cell specification and production, and on programmed cell death (PCD) [25]. Examinations of cell number before the onset of PCD, however, showed that strains with large numbers of ganglion cells as adults generally had equally large numbers of immature cells, and vice versa, suggesting that the process of cell death was not affected by this QTL.

### Summary of candidate genes and their relevance to retinal ganglion cell death and neurodegeneration

**Figure 4:**
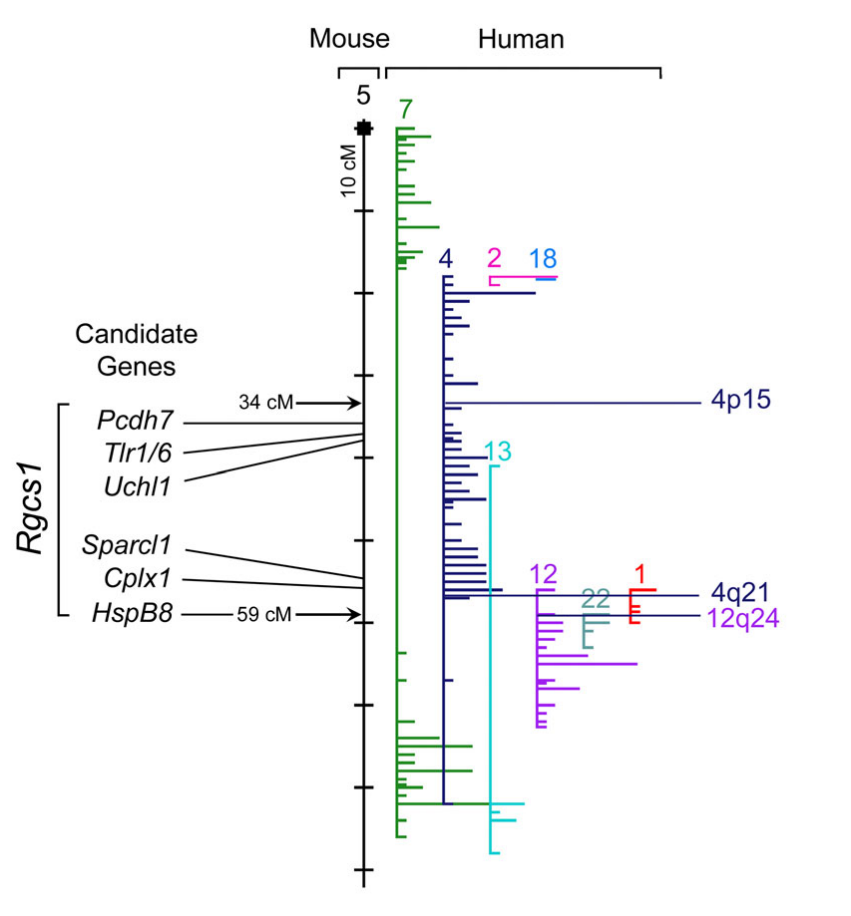
**Synteny map of *Rgcs1 *between mouse and human genomes**. The region of *Rgcs1 *on mouse chromosome 5 is shown along with the relative positions of the 7 candidate genes. These genes form 2 distinct clusters near the ends of the locus. The majority of the *Rgcs1 *locus is represented on human chromosome 4, between 4p15 and 4q21, although a small segment containing the human *HSPB8 *gene is located on chromosome 12 at 12q24.

The 7 candidate genes (Table 2) are distributed in two main clusters, each at either end of the 25 cM interval currently defining the *Rgcs1 *locus (Fig. 4). In response to a lesion of the optic nerve, each candidate gene could realistically play a role in the process of retinal ganglion cell death. However, this does not preclude the possibility that a different gene (or genes) in this region may be responsible for the QTL. Also, it is important to note that our evaluation of candidate genes did not address the possibility that the causative gene could be an expression QTL, where polymorphisms in the promoter region could affect expression levels. Alternatively, non-coding polymorphisms could also create alternative splice variants in some genes that can affect protein structure or transcript stability. Lastly, our *in silico *analysis did not address the possibilities of differences in non-coding RNAs (such as microRNAs) that may exist between the two strains, but which could also affect gene expression and cellular processes. Still, the filters we used for selection criteria were biased towards assessing relevant features that could dramatically account for a QTL between the two strains. A summary of each gene follows:

#### *Pcdh7 *(Protocadherin 7)

*Pcdh7 *is one of a member of a family of cadherin like molecules that play a specialized role in cell-cell interactions within the central nervous system [26,27]. The functional role of *Pcdh7 *is not well understood, but splice variants have been implicated in altering cell adhesion properties. This may affect both ganglion cell and retinal glial cell gene expression profiles by modifying cellular interactions with the extracellular matrix.

#### *Tlr1 *and *Tlr6 *(Toll-like Receptors 1 and 6)

There are 9 known *Tlr *genes in mice and all of them are expressed by microglia in the CNS [28]. In addition, indirect evidence suggests that astrocytes also express at least some *Tlr *genes. They function by allowing microglia to recognize and respond to a wide variety of pathogens and injury-resulting antigens, thus implicating these cells in both the innate and adaptive immune response of the CNS. It is not known if TLRs play a role in the activation response of microglia after optic nerve injury, but through TLR activation [28], these cells have been shown to express MHC class II proteins and act as antigen presenting cells in models of demyelinating diseases in mice [29]. Genetically controlled selective autoimmunity responses in mice can modulate the severity of retinal damage in acute (crush) and chronic (experimental glaucoma) models of optic nerve lesion [30-32]. This, along with the growing evidence that neuroinflammation can affect both neuronal survival and death [33], makes these genes attractive candidates for the cell death phenotype.

#### *Uchl1 *(ubiquitin C-terminal hydrolase-1)

UCHL-1 plays an integral role in the ubiquitin proteosome pathway. It hydrolyses small ubiquitin C-terminal adducts and may have a role in regulating the degradation of free ubiquitin monomers. Studies have documented 2 separate polymorphisms in human *UCHL-1 *that associate with either increased or decreased susceptibility to Parkinson's disease, although the concordance of these studies is variable. The pathology of *UCHL-1 *is most likely linked to changes in activity or expression levels. The putative Parkinson's disease causing allele in *UCHL-1 *results in a 50% decrease in enzyme catalytic activity [34]. Similarly, reduced overall gene expression of *UCHL-1 *is associated with the formation of Lewy bodies in patients with dementia [35] and the development of gracile axonal dystrophy and intraneuronal inclusions in mice [36]. These observations suggest that *Uchl1 *expression levels may play a factor in neuronal susceptibility. In addition to its suspected role as a susceptibitily allele for chronic neurodegeneration, *Uchl1 *expression is highly enriched in the ganglion cell layer. Although not polymorphic, quantitative differences in expression, if any, could account for the different cell death phenotypes between strains.

#### *Sparcl1 *(secreted protein acidic and rich in cysteine-like 1, *Hevin*, *SC1*)

The *Sparcl1 *gene product is a secreted protein that acts as a de-adhesive molecule [37]. It is expressed and secreted by neurons and glia presumably to allow cells to release contact from the extracellular matrix and begin to migrate. Increased *Sparcl1 *expression is associated with regions of neuronal injury, possibly playing a role in the glial activation response. *Sparcl1 *is the only gene we have identified that is enriched in the ganglion cell layer and has a polymorphism that results in a non-conservative amino acid change (an R to Q at position 384, near the junction of the acidic N-terminal domain and the Follistatin like domain [38]).

#### *Cplx1 *(Complexin I)

Complexin I is involved in synaptic vesicle trafficking in neurons. Complexins associate with the vesicle SNARE complex at some point during or just after the process of Ca^2+^-dependent fast synchronous transmitter release [39]. Both Complexin I and II have been implicated in human neurodegenerative disorders, but this involvement is not clear. The increased expression of both molecules has been documented after traumatic brain injury, suggesting involvement in the glutamate release response of damaged neurons [40]. Mice mutant for *Cplx1 *develop severe ataxia and behavioral disorders [41]. This gene was principally identified because it is highly expressed in the ganglion cell layer. However, its potential role in excitotoxic damage may affect ganglion cell death in both optic nerve crush and glaucoma insults, where secondary degeneration mediated by glutamate-toxicity has been implicated in both models of damage [42,43].

#### *HspB8 *(22 kDa heat shock protein)

HSPB8 is a member of the small heat shock protein (sHSP) family of molecular chaperones [44]. Mutations in the α-crystallin domain of *HSPB8 *in humans leads to distal hereditary motor neuropathy [45], a disease characterized by the degenerative loss of motor neurons. There have been reports that several members of the sHSP gene family are differentially regulated in animal models of glaucoma [46], although no study has focused specifically on *HspB8*.

### Rgcs1 and retinal ganglion cell death in glaucoma

Although *Rgcs1 *affects ganglion cell death after acute optic nerve crush, the role of the causative gene in this region as a susceptibility allele in glaucoma is not yet resolved. Acute nerve lesion and elevated IOP appear to activate a similar intrisinc apoptotic program, associated with *Bax*-dependent mitochondrial changes [14,15,47] and the activation of the caspase cascade [17,48-51]. Additionally, genome wide microarray studies conducted on early, intermediate, and late retinal gene expression changes from both axotomy injured rat eyes and eyes with experimental glaucoma showed significant overlap in the genes that were differentially expressed in both conditions [22]. These changes included early injury response genes (i.e., *cJun *and *Junb*), and stress response genes (i.e., *Hsp27 *and Ceruloplasmin). Additionally, ganglion cell specific genes, such as *Thy1 *and *Sncg*, were similarly down-regulated. There were specific differences in the pattern of differentially regulated genes, however, that may reflect different mechanisms of ganglion cell pathology between the two modalities of injury. Most prevalent was the up-regulation of genes involved in neuroinflammation, such as components of the complement cascade, in the experimental glaucoma retinas. Thus, neurinflammation may play a greater role in ganglion cell death during a chronic neurodegenerative process, than in an acute paradigm.

Interestingly, DBA/2J mice, which carry the resistant *Rgcs1 *genotype, exhibit a glaucoma phenotype characterized by progressive ganglion cell loss [52-54]. Since the dominant *Rgcs1 *allele does not completely abrogate cell death, we would not expect this strain to be completely resistant to glaucoma. Instead, we speculate that DBA/2J mice would exhibit a more severe phenotype if they carried the BALB/cByJ allele and experiments to create such a congenic strain are underway.

The role of *Rgcs1 *in glaucoma may also be inferred by comparison of mouse locus with the syntenic region in the human genome. The interval of interest on mouse Chr5 is principally represented on human Chr4 between 4p15 and 4q21 and a short segment of human Chr12, centered at 12q24 (Fig. 4). To date, no linkage studies examining forms of glaucoma with Mendelian inheritance patterns have identified genes or loci in these regions [8,55]. This is not surprising since these studies have depended on relatively rare forms of glaucoma, while most forms of primary open angle glaucoma exhibit much higher levels of genetic complexity [8]. We predict that the retinal ganglion cell susceptibility allele, if it does influence cell loss in glaucoma, would be just one of several genes that contribute to a more complex genetic disorder.

Interestingly, markers on human Chr4 that map near the region of interest (Fig. 4), showed promising results in a genome-wide screen of DNA from 113 sib-pairs affected with primary open angle glaucoma [56]. In particular, marker D4S2397 (4p15.2) was initially positive in 3 independent analyses, including model-dependent LOD scores and sib-pair maximum LOD scores. Marker D4S400 (4q21.22) was positive in 2 of 3 analyses. These markers on Chr4, however, failed to continue to show positive results when this analysis was expanded to include a second cohort of affected sib-pairs. Given the small size of this sib-pair screen, it may not yet be possible to interpret the relative importance of this region in human glaucoma, but it is encouraging that it has shown some promising results in linkage studies.

## Conclusion

Susceptibility of retinal ganglion cell death to a crush lesion of the optic nerve is influenced by genetic background. Linkage analysis of a large mapping population has helped resolve this genetic contribution to a locus on chromosome 5 between 34–59 cM. *In silico *examination of this region has identified 7 potential candidate genes that may account for the difference in ganglion cell death phenotype exhibited by the DBA/2J and BALB/cByJ strains. Evaluation of the effect of the BALB/cByJ locus in the DBA/2J mouse model of glaucoma will help confirm this gene/region as being a susceptibility allele for glaucoma as well as for acute optic nerve crush.

## Methods

### Animals

All mice were handled in accordance with the Association for Research in Vision and Ophthalmology statement for the use of animals for research and experimental protocols were approved by the Animal Care and Use Committee of the University of Wisconsin. DBA/2J and BALB/cByJ parental mice were purchased from the Jackson Laboratory (Bar Harbor, ME). These mice were bred to generate F1 offspring, which were then interbred by reciprocal crosses to generate F2 mice for analysis. Mice were housed in microisolator cages and kept on a 12 hr light/dark cycle. They were maintained on a 4% fat diet (8604 M/R, Harland Teklad, Madison, WI).

### Phenotyping and genotyping

To estimate the size of the mapping population required to successfully identify loci associated with the cell death phenotype, we conducted power calculations using phenotype and inheritance information acquired from earlier studies of smaller populations of N2 backcrossed and F2 mice [20]. The mouse genome size was approximated as 20 chromosomes of 100 cM in length and the proposed mapping interval was 20 cM. Since parental strain data provided no information regarding the dominance effect (δ), power calculations were made using the assumptions that δ = α (where α = the additive effect of phenotype) or δ = 0 (δ had no additive effect). Heritability of the locus of interest (*h*^2^) was then calculated using the information provided from our preliminary data sets (*h*^2 ^= 32.7% for δ = α, and 24.5% for δ = 0). Power calculations were performed using the formula and parameters described previously [57]. Within the heritability range of our existing population studies, these calculations suggested that a population of 100 F2 mice would have sufficient power to detect a single locus that associates with phenotype using this mapping strategy. To further increase the likelihood of detecting a QTL, we also biased the genotyping of the population to the most extreme ends of the distribution [58]. Consequently, 196 mice were phenotyped and from this, 60 were selected as the most resistant and 49 were selected as the most susceptible.

Consistent with our previous studies on other inbred mice, we aged the F2 pups to 8 weeks, at which time they underwent the optic nerve crush protocol. Optic nerve crush was performed on the left eye of each mouse using an intraorbital approach [20,59]. Previously, mice were euthanized 2 weeks after this procedure. However, time course analysis of cell loss over a 3 week period showed the same difference in cell loss between the 2 parental strains at both 2 and 3 weeks (data not shown). Since 3 weeks represented a more end-stage point of the cell death process, we opted to analyze the mapping population at this time after crush. Retinas were processed for quantification as described previously [20]. Approximately 10% of the entire population of neurons in the ganglion cell layer was sampled from each retina. Cell loss for each mouse was recorded as the percentage of cells remaining in the experimental retina relative to the fellow control retina. Previous studies have verified that DBA/2J and BALB/cByJ mice have an equal percentage of ganglion cells making up the neuronal population of the ganglion cell layer [20], and we have made the assumption that this relationship holds true to F2 mice generated from these parental strains.

More than 100 informative microsatellite markers between DBA/2J and BALB/cByJ mice were identified from the Mouse Genome Informatics (MGI) website of the Jackson Laboratory (please see Availability & requirements for more information). These markers were spaced roughly 20 cM apart and exhibited at least a 5 bp difference between the strains. Primer pairs for each marker were individually tested on genomic DNA isolated from spleens of parental mice. PCR conditions were empirically optimized for each primer pair, but typically primers were annealed at 55°C and reactions were run for a minimum of 40 cycles. Bands were evaluated on standard 1–3% agarose gels stained with ethidium bromide. After empirical testing, we selected 65 markers for genotyping. This included a minimum of 3 markers per chromosome and an approximate genome-wide scan radius of 15 cM with each marker separated by approximately 30 cM. At this distance, the proximal ends of chromosomes 1, 9, and 13, and the distal ends of chromosomes 3, 5, 11, and 19 were not fully covered by the markers used. Genotyping was performed using spleen DNA isolated from 60 resistant and 49 susceptible mice. Data were scored by an observer (JAD) masked to the phenotype and entered into an Excel (Microsoft, Redmond, WA) spreadsheet. Discrepancies in genotype reading were resolved by regenotyping.

### Data analysis

The resulting data set (of phenotype and genotype for all 109 mice) was analyzed using the R/qtl statistical software package (please see Availability & requirements for more information) [60]. Simple interval mapping adjusting for possible sex by genotype interaction was performed across the genome with 2cM steps. A 5% permutation threshold [61] was estimated as 4.15 based on 10000 sets of permuted genotypes.

### In silico analysis to identify candidate genes

Chromosomal regions of interest identified from interval mapping analysis were analyzed further for potential candidate genes using an *in silico *approach. All known genes and ESTs present in the region were identified from mouse genomic sequence data present in the MGI database (please see Availability & requirements for more information). This database also included supplementary information on localization and known function of gene products. Candidate genes were identified using three levels of criteria.

#### Criterion 1: SNP analysis

Because there is sequence data available for multiple strains, it was possible to data mine for SNPs between DBA/2J and BALB/cByJ mice in the region of interest, including SNPs that create non-synonymous amino acid changes. Informative SNPs could also be examined for strain clustering, when possible, using data on the cell death phenotype we had characterized for other mouse strains [20]. Reference strains included 129X1/SvJ and C57BL/6J as resistant strains, and C3H/HeJ and NOD/LtJ as susceptible strains. Intermediate strains included A/J (likely resistant) and NZB/BINJ (likely susceptible). SNPs that were negative for strain clustering (i.e., DBA/2J and NOD/LtJ mice sharing the same polymorphism, etc) were excluded from further consideration.

#### Criterion 2: microarray expression data

The list of genes identified in a region of interest was also compared to gene expression profiles reported for mammalian retinal ganglion cells, the ganglion cell layer, or whole retina in response to glaucoma or optic nerve lesion. These profiles included: (i) laser captured human ganglion cell layer [62], (ii) primary cultures of purified rat retinal ganglion cells [63,64], (iii) non-human primate retina with experimental glaucoma [65], (iv) rat retina with experimental glaucoma or after axotomy [22], (v) rat optic nerve heads with experimental glaucoma [66], (vi) and aging retinas from DBA/2J mice [46].

#### Criterion 3: Functional analysis

Finally, genes in this region were screened for functional information that indicated their expression pattern and linked them as susceptibility alleles in other neurodegenerative diseases. Some genes for which there was no expression data confirming expression in the CNS, or any known functional data, were not considered for selection in this analysis even though they may have met other criteria.

## Availability & requirements

Genomic sequence information: 

Mouse Genome Informatics (MGI) website of the Jackson Laboratory: 

R/qtl statistical software package: 

MGI database: 

## Authors' contributions

RWN (corresponding author) conceived of the study, directed the research, and wrote the manuscript. YL did all the optic nerve crush surgeries and phenotyped cell loss in the F2 mice. JAD isolated genomic DNA, conducted microsatellite PCRs on the F2 mice selected for genotyping, prepared data for statistical analysis, and helped perform the *in silico *examination of chromosome 5. LMC and BSY performed all statistical analysis of the linkage data. CLS contributed to the overall experimental design, development of the mapping population and data acquisition, and preparation of the manuscript. All authors read and approved the final manuscript.

## References

[B1] Quigley HA, Enger C, Katz J, Sommer A, Scott R, Gilbert D (1994). Risk factors for the development of glaucomatous visual field loss in ocular hypertension. Arch Ophthalmol.

[B2] Kass MA, Heuer DK, Higginbotham EJ, Johnson CA, Keltner JL, Miller JP, Parrish II RK, Wilson MR, Gordon MO (2002). The ocular hypertension study: A randomized trial determines that topical ocular hypertensive medication delays or prevents the onset of primary open-angle glaucoma. Arch Ophthalmol.

[B3] Heijl A, Leske MC, Bengtsson B, Hyman L, Bengtsson B, Hussein M (2002). Reduction of intraocular pressure and glaucoma progression. Arch Ophthalmol.

[B4] Burgoyne CF, Downs JC, Bellezza AJ, Suh JK, Hart RT (2005). The optic nerve head as a biomechanical structure: a new paradigm for understanding the role of IOP-related stress and strain in the pathophysiology of glaucomatous optic nerve head damage. Prog Retin Eye Res.

[B5] Ethier CR (2006). Scleral biomechanics and glaucoma - a connection?. Can J Ophthalmol.

[B6] Whitmore AV, Libby RT, John SWM (2005). Glaucoma: Thinking in new ways - a role for autonomous axonal self-destruction and compartmentalised processes?. Prog Retin Eye Res.

[B7] Nickells RW (2007). From ocular hypertension to ganglion cell death: a theoretical sequence of events leading to glaucoma. Can J Ophthalmol.

[B8] Wiggs JL (2007). Genetic etiologies of glaucoma. Arch Ophthalmol.

[B9] Tielsch JM, Katz J, Sommer A, Quigley HA, Javitt JC (1994). Family history and risk of primary open angle glaucoma: The Baltimore Eye Survey. Arch Ophthalmol.

[B10] Klein BEK, Klein R, Lee KE (2004). Heritability of risk factors for primary open angle glaucoma: The Beaver Dam Eye Study. Invest Ophthalmol Vis Sci.

[B11] Cook R, Lu L, Gu J, Williams RW, Smeyne RJ (2003). Identification of a single QTL, Mptp1, for susceptibility to MPTP-induced substantia nigra pars compacta neuron loss in mice. Mol Brain Res.

[B12] Sedelis M, Hofele K, Schwarting RKW, JHuston JP, Belknap JK (2003). Chromosomal loci influencing the susceptibility to the Parkinsonian neurotoxin 1-methyl-4-phenyl-1,2,3,6-tetrahydropyridine. J Neurosci.

[B13] Lorenzana A, Chancer Z, Schauwecker PE (2007). A quantitative trait locus on chromosome 18 is a critical determinant of excitotoxic cell death susceptibility. Eur J Neuro.

[B14] Li Y, Schlamp CL, Poulsen KP, Nickells RW (2000). Bax-dependent and independent pathways of retinal ganglion cell death induced by different damaging stimuli. Exp Eye Res.

[B15] Libby RT, Li Y, Savinova OV, Barter J, Smith RS, Nickells RW, John SWM (2005). Susceptibility to neurodegeneration in glaucoma is modified by Bax gene dosage. PLoS Genet.

[B16] Schlamp CL, Johnson EC, Li Y, Morrison JC, Nickells RW (2001). Changes in Thy1 gene expression associated with damaged retinal ganglion cells. Mol Vis.

[B17] McKinnon SJ, Lenhman DM, Kerrigan-Baumrind LA, Merges CA, Pease ME, Kerrigan DF, Ransom NL, Tahzib NG, Reitsamer HA, Levkovitch-Verbin H, Quigley HA, Zack DJ (2002). Caspase activation and amyloid precursor protein cleavage in rat ocular hypertension. Invest Ophthalmol Vis Sci.

[B18] Huang W, Fileta JB, Dobberfuhl A, Filippopoulos T, Guo Y, Kwon G, Grosskreutz CL (2005). Calcineurin cleavage is triggered by elevated intraocular pressure, and calcineurin inhibition blocks retinal ganglion cell death in experimental glaucoma. Proc Natl Acad Sci USA.

[B19] Huang W, Fileta J, Guo Y, Grosskreutz CL (2006). Downregulation of Thy1 in retinal ganglion cells in experimental glaucoma. Curr Eye Res.

[B20] Li Y, Semaan SJ, Schlamp CL, Nickells RW (2007). Dominant inheritance of retinal ganglion cell resistance to optic nerve crush in mice. BMC Neurosci.

[B21] Manichaikul A, Dupuis J, Sen A, Broman KW (2006). Poor performance of bootstrap confidence intervals for the location of a Quantitative Trait Locus. Genetics.

[B22] Yang Z, Quigley HA, Pease ME, Yang Y, Qian J, Valenta D, Zack DJ (2007). Changes in gene expression in experimental glaucoma and optic nerve transection: the equilibrium between protective and detrimental mechanisms. Invest Ophthalmol Vis Sci.

[B23] Ferraro TN, Smith GG, Schwebel CL, Lohoff FW, Furlong P, Berrettini WH, Buono RJ (2007). Quantitative trait locus for seizure susceptibility on mouse chromosome 5 confirmed with reciprocal congenic strains. Physiol Genomics.

[B24] Williams RW, Strom RC, Goldwitz D (1998). Natural variation in neuron number in mice is linked to a major quantitative trait locus on Chr 11. J Neurosci.

[B25] Strom RC, Williams RW (1998). Cell production and cell death in the generation of variation in neuron number. J Neurosci.

[B26] Yoshida K, Watanabe M, Kato H, Dutta A, Sugano S (1999). BH-protocadherin-c, a member of the cadherin superfamily, interacts with protein phosphatase 1 alpha through its intracelular domain. FEBS Lett.

[B27] Yoshida K (2003). Fibroblast cell shape and adhesion *in vitro* is altered by overexpression of the 7a and 7b isoforms of protocadherin 7, but not the 7c isoform. Cell Mol Biol Lett.

[B28] Olson JK, Miller SD (2004). Microglia initiate central nervous system innate and adaptive immune responses through mulitple TLRs. J Immunol.

[B29] Aloisi F, Ria F, Adorini L (2000). Regulation of T-cell responses by CNS antigen-presenting cells: different roles for microglia and astrocytes. Immunol Today.

[B30] Fisher J, Levkovitch-Verbin H, Schori H, Yoles E, Butovsky O, Kaye FJ, Ben-Nun A, Schwartz M (2001). Vaccinations for neuroprotection in the mouse optic nerve: implications for optic neuropathies. J Neurosci.

[B31] Kipnis J, Yoles E, Schori H, Hauben E, Shaked I, Schwartz M (2001). Neuronal survival after CNS injury is determined by a genetically encoded autoimmune response. J Neurosci.

[B32] Bakalash S, Shlomo GB, Aloni E, Shaked I, Wheeler LA, Ofri R, Schwartz M (2005). T-cell-based vaccination for morphological and functional neuroprotection in a rat model of chronically elevated intraocular pressure. J Mol Med.

[B33] Berliocchi L, Corrasaniti MT, Bagetta G, Lipton SA (2007). Neuroinflammation in neuronal degeneration and repair. Cell Death Differ.

[B34] Healy DG, Abou-Sleiman PM, Wood NW (2004). Genetic causes of Parkinson's disease: UCHL-1. Cell Tissue Res.

[B35] Barrachina M, Castano E, Dalfo E, Maes T, Buesa C, Ferrer I (2006). Reduced ubiquitin C-terminal hydrolase-1 expression levels in dementia with Lewy bodies. Neurobiol Dis.

[B36] Saigoh K, Wang YL, Suh JG, Yamanishi T, Sakai Y, Kiyosawa H, Harada T, Ichihara N, Wakana S, Kikuchi T, Wada K (1999). Intragenic deletion in the gene encoding ubiquitin carboxy-terminal hydrolase in gad mice. Nat Genet.

[B37] Girard JP, Springer TA (1996). Modulation of endothelial cell adhesion by Hevin, an acidic protein associated with High Endothelial Venules. J Biol Chem.

[B38] Sullivan MM, Sage EH (2004). Hevin/SC1, a matricellular glycoprotein and potential tumor-suppressor of the SPARC/BM-40/Osteonectin family. Int J Biochem Cell Biol.

[B39] Hong W (2005). SNAREs and traffic. Biochim Biophys Acta.

[B40] Yi JH, Hoover R, McIntosh TK, Hazell AS (2006). Early, transient increase in Complexin I and Complexin II in the cerebral cortex following traumatic brain injury is attenuated by N-acetylcysteine. J Neurotrama.

[B41] Glynn D, Drew CJ, Reim K, Brose N, Morton AJ (2005). Profound ataxia in complexin I knockout mice masks a complex phenotype that includes exploratory and habituation deficits. Hum Mol Genet.

[B42] Yoles E, Schwartz M (1998). Elevation of intraocular glutamate levels in rats with partial lesion of the optic nerve. Arch Ophthalmol.

[B43] Dreyer EB, Zurakowski D, Schumer RA, Podos SM, Lipton SA (1996). Elevated glutamate levels in the vitreous body of humans and monkeys with glaucoma. Arch Ophthalmol.

[B44] Carra S, Sivilotti M, Zobel ATC, Lambert H, Landry J (2005). HspB8, a small heat shock protein mutated in human neuromuscular disorders, has in vivo chaperone activity in cultured cells. Hum Mol Genet.

[B45] Benndorf R, Welsh MJ (2004). Shocking degeneration. Nat Genet.

[B46] Steele MR, Inman DM, Calkins DJ, Horner PJ, Vetter ML (2006). Microarray analysis of retinal gene expression in the DBA/2J model of glaucoma. Invest Ophthalmol Vis Sci.

[B47] Mittag TW, Danias J, Pohorenec G, Yuan HM, Burakgazi E, Chalmers-Redman R, Podos SM, Tatton WG (2000). Retinal damage after 3 to 4 months of elevated intraocular pressure in a rat glaucoma model. Invest Ophthalmol Vis Sci.

[B48] Kermer P, Ankerhold R, Klocker N, Krajewski S, Reed JC, Bähr M (2000). Caspase-9 involvement in secondary death of axotomized rat retinal ganglion cells in vivo. Brain Res Mol Brain Res.

[B49] Kermer P, Klocker N, Labes M, Thomsen S, Srinivasan A, Bähr M (1999). Activation of caspase-3 in axotomized rat retinal ganglion cells in vivo. FEBS Lett.

[B50] Weishaupt JH, Diem R, Kermer P, Krajewski S, Reed JC, Bahr M (2003). Contribution of caspase-8 to apoptosis of axotomized rat retinal ganglion cells *in vivo*. Neurobiology of Disease.

[B51] Huang W, Dobberfuhl A, Filippopoulos T, Ingelsson M, Fileta JB, Poulin NR, Grosskreutz CL (2005). Transcriptional up-regulation and activation of initiating caspases in experimental glaucoma. Am J Pathol.

[B52] Libby RT, Anderson MG, Pang IH, Robinson ZH, Savinova OV, Cosma IM, Snow A, Wilson LA, Smith RS, Clark AF, John SWM (2005). Inherited glaucoma in DBA/2J mice: pertinent disease features for studying the neurodegeneration. Vis Neurosci.

[B53] Danias J, Lee KC, Zamora MF, Chen B, Shen F, Filippopoulos T, Su Y, Goldblum D, Podos SM, Mittag T (2003). Quantitative analysis of retinal ganglion cell (RGC) loss in aging DBA/2NNia glaucomatous mice: comparison with RGC loss in aging C57BL/6 mice. Invest Ophthalmol Vis Sci.

[B54] Schlamp CL, Li Y, Dietz JA, Janssen KT, Nickells RW (2006). Progressive ganglion cell loss and optic nerve degeneration in DBA/2J mice is variable and asymmetric. BMC Neurosci.

[B55] Libby RT, Gould DB, Anderson MG, John SWM (2005). Complex genetics of glaucoma susceptibility. Ann Rev Genomics Hum Genet.

[B56] Wiggs JL, Allingham RR, Hossain A, Kern J, Auguste J, DelBono EA, Broomer B, Graham FL, Hauser M, Pericak-Vance M, Haines JL (2000). Genome-wide scan for adult onset primary open angle glaucoma. Hum Mol Genet.

[B57] Dupuis J, Siegmund D (1999). Statistical methods for mapping quantitative trait loci from a dense set of markers. Genetics.

[B58] Darvasi A, Soller M (1992). Selective genotyping for determination of linkage between a marker locus and a quantitative locus. Theor App Genet.

[B59] Li Y, Schlamp CL, Nickells RW (1999). Experimental induction of retinal ganglion cell death in adult mice. Invest Ophthalmol Vis Sci.

[B60] Broman KW, Wu H, Sen AS, Chruchill GA (2003). R/qtl: QTL mapping in experimental crosses. Bioinformatics.

[B61] Churchill GA, Doerge RW (1994). Empirical threshold values for quantitative trait mapping. Genetics.

[B62] Kim CY, Kuehn MH, Clark AF, Kwon YH (2006). Gene expression profile of the adult human retinal ganglion cell layer. Mol Vis.

[B63] Farkas RH, Qian J, Goldberg JL, Quigley HA, Zack DJ (2004). Gene expression profiling of purified rat retinal ganglion cells. Invest Ophthalmol Vis Sci.

[B64] Ivanov D, Dvoriantchikova G, Nathanson L, McKinnon SJ, Shestopalov VI (2006). Microarray analysis of gene expression in adult retinal ganglion cells. FEBS Lett.

[B65] Miyahara T, Kikuchi T, Akimoto M, Kurokawa T, Shibuki H, Yoshimura N (2003). Gene microarray analysis of experimental glaucomatous retina from Cynomologous monkey. Invest Ophthalmol Vis Sci.

[B66] Johnson EC, Jia L, Cepurna WA, Doser TA, Morrison JC (2007). Global changes in optic nerve head gene expression after exposure to elevated intraocular pressure in a rat glaucoma model. Invest Ophthalmol Vis Sci.

